# Peroxisome proliferator-activated receptor-γ as the gatekeeper of tight junction in *Clostridioides difficile* infection

**DOI:** 10.3389/fmicb.2022.986457

**Published:** 2022-11-11

**Authors:** Yi-Hsin Lai, Tai-Chieh Wu, Bo-Yang Tsai, Yuan-Pin Hung, Hsiao-Ju Lin, Yau-Sheng Tsai, Wen-Chien Ko, Pei-Jane Tsai

**Affiliations:** ^1^Institute of Basic Medical Sciences, College of Medicine, National Cheng Kung University, Tainan, Taiwan; ^2^Department of Medical Laboratory Science and Biotechnology, College of Medicine, National Cheng Kung University, Tainan, Taiwan; ^3^Departments of Internal Medicine, Tainan Hospital, Ministry of Health & Welfare, Tainan, Taiwan; ^4^Department of Internal Medicine, National Cheng Kung University Hospital, College of Medicine, National Cheng Kung University, Tainan, Taiwan; ^5^Department of Medicine, National Cheng Kung University, Tainan, Taiwan; ^6^Institute of Clinical Medicine, National Cheng Kung University, Tainan, Taiwan; ^7^Clinical Medicine Research Center, National Cheng Kung University Hospital, Tainan, Taiwan; ^8^Center of Infectious Disease and Signaling Research, National Cheng Kung University, Tainan, Taiwan; ^9^Department of Pathology, National Cheng Kung University Hospital, College of Medicine, National Cheng Kung University, Tainan, Taiwan

**Keywords:** *Clostridioides difficile*, peroxisome proliferator-activated receptor-γ, tight junction, occludin, pioglitazone

## Abstract

*Clostridioides difficile* is a major causative pathogen of nosocomial antibiotic-associated diarrhea and severe colitis. Despite the use of vancomycin and fidaxomicin as standard drugs for the treatment of *C. difficile* infection (CDI), clinical relapse rates remain high. Therefore, new alternative therapeutics to treat CDI are urgently required. The nuclear receptor, peroxisome proliferator-activated receptor-γ (PPAR-γ), is mainly expressed in the adipose tissue and modulates lipid metabolism and insulin sensitization. Previous studies have shown that PPAR-γ is highly expressed in colonic tissues and regulates tight junction function in epithelial cells. However, the role of PPAR-γ in CDI pathogenesis remains unclear. In this study, we used a mouse model of CDI and found that both expression levels of PPAR-γ and the tight junction protein, occludin, were decreased in colonic tissues. Furthermore, to investigate the role of PPAR-γ in CDI, we used PPAR-γ defective mice and found that intestinal permeability and bacterial dissemination in these mice were significantly higher than those in wild-type mice during CDI. Administration of the PPAR-γ agonist, pioglitazone, to activate PPAR-γ activity improved the phenotypes of CDI, including bodyweight loss, inflammation, and intestinal integrity. Taken together, these results demonstrate that PPAR-γ is a potential therapeutic target in CDI, as it modulates colonic inflammation and integrity.

## Introduction

*Clostridioides difficile* is a spore-forming Gram-positive anaerobic bacterium. *Clostridioides difficile* infection (CDI) may be asymptomatic or result in diarrhea, pseudomembranous colitis, and toxic megacolon; it can even lead to death (Gil et al., 2018). Toxins A and B, encoded by the genes *tcdA* and *tcdB,* respectively, are the main virulent factors in *C. difficile* that cause disease (Di Bella et al., 2016; Mileto et al., 2019). After ingestion, *C. difficile* spores germinate into vegetative cells in small intestine. *Clostridioides difficile* can colonize the colon when the intestinal microbiota is disrupted by antimicrobial therapy (Kelly and LaMont, 2008; Paredes-Sabja et al., 2014). Toxigenic *C. difficile* strains, which produce toxin A, B, or both, impair the epithelial barrier and alter the cytoskeletal structure by glucosylating Rho family proteins (Kelly and LaMont, 2008; Chen et al., 2015), thus stimulating colonic inflammation and triggering cytokine release (Gerhard et al., 2011; Cowardin et al., 2016). The disruption of gut epithelial barrier is essential for CDI.

Impaired host immunity resulting from different factors, such as advanced age, presence of comorbidity with functional impairment including inflammasome activation (Liu et al., 2018), or gene polymorphisms (such as Toll-like receptor 2/4, interleukin IL-8), is associated with increased risk of CDI and disease recurrence (Garey et al., 2010; Ryan et al., 2011; Lai et al., 2021; Simpson et al., 2022). Despite these findings, the main factor in protective immunity against CDI remains unclear. Current antibiotic therapies, including treatments with vancomycin and fidaxomicin, may alleviate the disease (Khanna et al., 2016; Cho et al., 2020). However, the emergence of antibiotic-resistant *C. difficile* strains and the risk of recurrent CDI (rCDI) after antibiotic treatment are major public health concerns (Cornely et al., 2012; Sholeh et al., 2020; Tsigrelis, 2020; Dilnessa et al., 2022). Although fecal microbiota transplantation is effective in treating rCDI, the efficacy, safety, and stability of this method need to be considered and discussed (Weingarden et al., 2015; Colleen et al., 2016; Youngster et al., 2016; Chiu et al., 2021).

Peroxisome proliferator-activated receptor-γ (PPAR-γ), mainly expressed in adipose tissues, belongs to the nuclear receptor family and is a transcription factor that controls gene expression in lipid metabolism, glucose homeostasis, and immune modulation (Dubuquoy et al., 2006; Straus and Glass, 2007; Mirza et al., 2019; Chen et al., 2021; Yang et al., 2021). In addition to adipose tissues, PPAR-γ is expressed in colonic tissues, where it is distributed in epithelial cells (ECs) and, to a lesser degree, in macrophages and lymphocytes (Dubuquoy et al., 2002; Shah et al., 2007; Su et al., 2007; Mohapatra et al., 2010). PPAR-γ expression is markedly reduced in colonic ECs in patients with ulcerative colitis (UC), a bowel inflammation disease (Dubuquoy et al., 2003; Dou et al., 2015). In a mouse model of dextran sodium sulfate-induced colitis, treatment with a thiazolidinedione, which is a PPAR-γ agonist, reduced colonic inflammation *via* NFκB-dependent (Takaki et al., 2006; Simeoli et al., 2016) and myosin light-chain kinase-dependent mechanisms (Zhao et al., 2018). A previous study has reported a link between PPAR-γ and colonic inflammation in CDI by establishing an animal model of CDI using wild-type (WT) mice and T cell-specific PPAR-γ null mice, and showed that the loss of PPAR-γ in T cells increased disease severity and colonic inflammation in the mice with CDI (Viladomiu et al., 2012). However, the role of stromal PPAR-γ in CDI remains unclear.

During CDI, there is loss of integrity of gut epithelial cells and damage to gut tissue. Therefore, therapeutic interventions capable of restoring the intestinal epithelial barrier are imperative and warrant further investigations. Growing evidence suggest PPAR-γ controls not only the inflammatory response but also the barrier function of epithelial and endothelial cells (Ogasawara et al., 2010). Activation of PPAR-γ enhances the barrier function and upregulates tight junction protein expression in intestinal ECs, urothelial cells, nasal ECs, brain endothelial cells, and pulmonary endothelial cells (Varley et al., 2006; Huang et al., 2009; Ogasawara et al., 2010; Li et al., 2014). Nevertheless, there is a lack of evidence supporting the relationship of PPAR-γ and tight junction proteins in the cases or animals of CDI. In this study, we utilized a murine model of CDI to investigate the mechanism by which *C. difficile* impairs the intestinal epithelial barrier and elucidate the molecular pathway that regulates the expression of tight junction proteins. In summary, we elucidate a mechanism underlying PPAR-γ-mediated repair of tight junctions, wherein increased PPAR-γ activity improves intestinal integrity and alleviates pathogenic effects.

## Materials and methods

### Bacterial strains

*Clostridioides difficile* JIK 8284 (*tcdA^+^, tcdB^+^*) and the isogenic mutant strain DLL 3121 (*tcdA^−^, tcdB^−^*) used in this study were kindly provided by Prof. Dena Lyras in Monash University, Australia. *Clostridioides difficile* strains were cultured anaerobically at 37°C on blood agar (BD Life Science, San Diego, CA) or in brain heart infusion broth (BHIS, BD Life Science, San Diego, *CA.*), supplemented with 5 mg/ml yeast extract (MO BIO Laboratories San Diego biotech corridor *CA.*) and 0.1% L-cysteine (AMRESCO^®^, Solon, United States). Isogenic mutant strains were maintained on CDC plates or in BHIS broth containing erythromycin (20 μg/ml) and lincomycin (20 μg/ml).

### *Clostridioides difficile* infection mouse model

Wild-type C57BL/6JNarl mice were obtained from the National Laboratory Animal Center in Tainan. Seven to eight-week-old (20–25 g) B6 mice were given a mixture of antibiotics (kanamycin, 0.4 mg/ml; gentamycin, 0.035 mg/ml; colistin, 0.057 mg/ml; metronidazole, 0.215 mg/ml; and vancomycin, 0.045 mg/ml) in the drinking water daily for 5 days before infection with C. difficile (i.e., from day −5 to −1). On the day before infection, mice were given esomeprazole (Nexium^®^, 40 mg/kg/day) by oral gavage. On day 0, mice were injected intraperitoneally with clindamycin (4 mg/kg) and then challenged orally with 3.5 × 107 colony forming units (CFUs) of vegetative C. difficile cells (JIK 8284; tcdA+, tcdB+). The vehicle group was challenged with phosphate-buffered saline (PBS) instead of *C. difficile*. Mice were weighed and symptoms of CDI were recorded daily, until they were sacrificed at 48 h post-infection. Mice were bred and housed in the animal facility of National Cheng Kung University. All animal studies were performed according to the protocols approved by the Institutional Animal Care and Use Committee of National Cheng Kung University (IACUC no.:105183, 105,181).

### Peroxisome proliferator-activated receptor-γ defective mice

A lack of PPAR-γ is lethal to embryonic mice. PPAR-γ defective (*Pparg^C/−^*) mice were utilized to examine the effect of PPAR-γ deficiency on clostridial colitis. The modification of gene locus carried by *Pparg^C/−^* mice has been described previously (Tsai et al., 2004). With the insertion of the AU-rich elements (ARE) in the 3′-untranslated region of c-fos transcripts into *Pparg* gene locus, we had generated a *Pparg^C/+^*mice with basal *Pparg* mRNA levels to 75% normal level. After breeding *Pparg*^*C*/+^ mice with *Pparg*^+/−^ mice, *Pparg^C/−^* pups were produced. The expression of *Pparg* mRNA in *Pparg^C/−^* mice was decreased to ~25% normal level, here *Pparg^C/−^* mice represented a hypomorphic PPAR-γ mouse model (Tsai et al., 2004; Liu et al., 2016).

### Phenotypic analysis of *Clostridioides difficile* infection

Reported signs of colitis in mice included weight loss, diarrhea, and death. Disease severity was scored by stool consistency and death, as follows: “0” indicates well-formed pellets; “1” indicates semiformed stools that did not adhere to the anus; “2” indicates semiformed stools that adhere to the anus; “3” indicates liquid stools; “4” indicates severe for perianal soiling, rectal bleeding, and diarrhea; and “5” indicates death. Thus, the changes in body weight, stool consistency, gross view of gut, and cecal weight were selected to estimate the severity of CDI in mice.

### Luminescent reporter mouse model

FVB/JNarl background NF-κB-RE-Luc reporter mice have been demonstrated in the CDI murine model previously (Hung et al., 2015). Eight to twelve-week-old (>25 g) luminescent reporter mice were administrated as previously described for the C57BL/6JNarl mouse model for 2 days before CDI. On the day of *C. difficile* challenge, mice were injected with clindamycin (4 mg/kg) intraperitoneally and then orogastrically challenged with 3.5 × 10^7^ CFUs of *C. difficile* vegetative bacteria. Mice in the experimental group were fed with different doses of pioglitazone (20, 40, or 70 mg/kg/day) for five days before *C. difficile* to sacrifice. Body weight and signs of CDI were recorded daily until mice were sacrificed 48 h after CDI.

### *In vivo* imaging system

NF-κB-dependent reporter mice were used to address the inflammatory extent based on the spatial and temporal pattern *in vivo*. Luciferin was injected intraperitoneally at 150 mg/kg body weight 10 min before imaging. Mice were anesthetized with isoflurane/oxygen, and images were collected for 5 min by IVIS^®^ Spectrum Imaging System (Xenogen Corp, Alameda, CA).

### Dextran-FITC translocation assay

Mice were challenged with dextran-fluorescein isothiocyanate (FITC; 500 mg/kg body weight, Sigma-Aldrich) by oral gavage after CDI and were fed nothing by mouth for 3 h thereafter. The amount of dextran-FITC in the serum collected *via* heart puncture was measured with a Modulus™ II Microplate Multimode Reader (Turner Biosystems, Sunnyvale, CA).

### Bacterial dissemination

After mice were sacrificed, liver, spleen, and kidney were excised, weighed, and homogenized with a homogenizer (MagNA Lyser Instrument, Roche Applied Science) aseptically. The suspension of homogenized tissues was plated on BAPs (blood agar plates) and anaerobic blood agar plates. These plates were incubated at 37°C in aerobic and anaerobic conditions to obtain bacterial counts. Bacterial dissemination incidence was calculated as a percentage of mice with bacterial-infected organs to total number of mice in the experiment.

### *In vitro* cell line infection

Human colorectal adenocarcinoma cell line (HT-29 cell) was used as an *in vitro* infection model. The cells were infected with a multiplicity of CDI (MOI 50) for 4 h under the condition of 37°C and 5% CO_2_. To estimate the effect of pioglitazone, cells were pre-treated with 20 or 40 μM pioglitazone for 24 h.

### xCELLigence system

The xCELLigence system (Roche Applied Science, Germany), known as the Real-Time Cell Analyzer (RTCA), has three components: an analyzer, a device station, and a 96-well E-plate. To evaluate the temporal change of cell tight junction function, 50 μl of the culture medium was added to the 96-well E-plate to obtain background readings. A volume of 200 μl of HT-29 cell suspension was added afterward. The E-plate containing 2 × 10^5^ cells was incubated overnight at 37°C, 5% CO_2_ in the incubator, and 20 μM pioglitazone or dimethyl sulfoxide (DMSO) was given 1 h before the addition of *C. difficile*. Impedance reflected by the cell index (CI) was recorded continuously. The final CI was compared to that before infection.

### Quantification of gene expression

RNA of HT-29 cells and mouse colonic tissues was extracted by combined REzol (Protech Technology, Taiwan) and total RNA mini kit (Geneaid Biotech Ltd., Taipei, Taiwan), according to the manufacturer’s instructions. Gene expression of PPAR-γ, occludin, Nrf1, and Tfam, was evaluated by real-time PCR.

### Immunoblotting

Homogenized mouse tissues and cultured HT-29 were collected and lysed with RIPA lysis buffer (50 mM Tris-base, 0.25% Sodium deoxycholate, 1% NP-40, 150 mM Sodium chloride, 1 mM EDTA). Proteins were determined by standard Bradford assay (Bio-Rad). Equivalent amounts of protein were loaded and separated by sodium dodecyl sulfate-polyacrylamide (SDS-PAGE) gel electrophoresis, then transferred onto polyvinylidene fluoride (PVDF) membranes (Pall Gelman Laboratory). After incubation with 5% non-fat milk or bovine serum albumin (BSA) in Tris-buffered saline with Tween 20 (TBST:10 mM Tris pH 8.0, 150 mM NaCl, and 1% Tween 20) for 1 h, membranes were washed three times with TBST and incubated overnight at 4°C with appropriate dilution of primary antibodies against PPAR-γ, occludin (Cell Signaling/ Invitrogen), glyceraldehyde 3-phosphate dehydrogenase (GAPDH), or ß-actin (Sigma-Aldrich). Membranes were washed and incubated for 1 h at room temperature with appropriate dilution of anti-rabbit (for PPAR-γ/occludin; Calbiochem) or anti-mouse (for GAPDH/ß-actin; Calbiochem) secondary antibody. Blots were washed and developed with the Enhanced Chemiluminescent (ECL) detection reagent (GE Healthcare), according to the manufacturer’s protocols.

### Immunofluorescence staining

Colonic tissues were collected and embedded in paraffin, sectioned (5 μm), deparaffinized, and rehydration. After antigen unmasking by boiling slides in sodium citrate solution, sections were blocked with StartingBlock™ blocking buffer T20 (PBS; Thermo Scientific™, United States). After overnight incubation at 4°C with rabbit anti-mouse occludin (Invitrogen) or PPAR-γ (Cell signaling) antibody, slides were washed with PBS-Tween 20 and incubated with goat Alexa-Fluor 488 anti-rabbit IgG (1:300, Invitrogen) for 1 h at room temperature. Nuclei were also stained with Hoechst (1:5,000) at room temperature for 5 min. Finally, sections were washed and examined with an Olympus microscope (Olympus^®^ DP73). Images were captured by the CellSens software (Olympus^®^). Images were analyzed using the TissueQuest software (TissueGnostics, Vienna, Austria). Using the gating feature of the TissueQuest software, nuclei were detected by dissection algorithms in the DAPI channel. Analysis was restricted to nuclear signals to obtain cell-based immunofluorescence results. The signals were plotted against the nuclear DAPI signals to create FACS-like scattergrams. Fluorescence intensity was analyzed as the percentage of the intensity using ImageJ software.

### Electron microscopic images

After mice were sacrificed, fresh colonic tissues were fixed with glutaraldehyde for 24 h (pH = 7.4, 4°C) and then dehydrated in ethanol. Thereafter, tissues were embedded in resin and further used on an ultramicrotome (Leica EM UC7, Leica Microsystems, Belgium) to prepare high-quality ultra-sections. Images of colonic tissues were viewed and imaged with transmission electron microscope (Hitachi HT-7700, Japan) in an acceleration potential of 120KeV.

### Statistical analyses

The data were calculated using the unpaired Student’s *t*-test. Statistical analysis and graphing were analyzed using GraphPad Prism version 6.0. Multiple group comparisons were accomplished by one-way ANOVA. Statistical significance was set at *p*-values of <0.05.

## Results

### Peroxisome proliferator-activated receptor-γ and tight junction proteins decreased in colonic tissues from mice *Clostridioides difficile* infection

**Figure 1:**
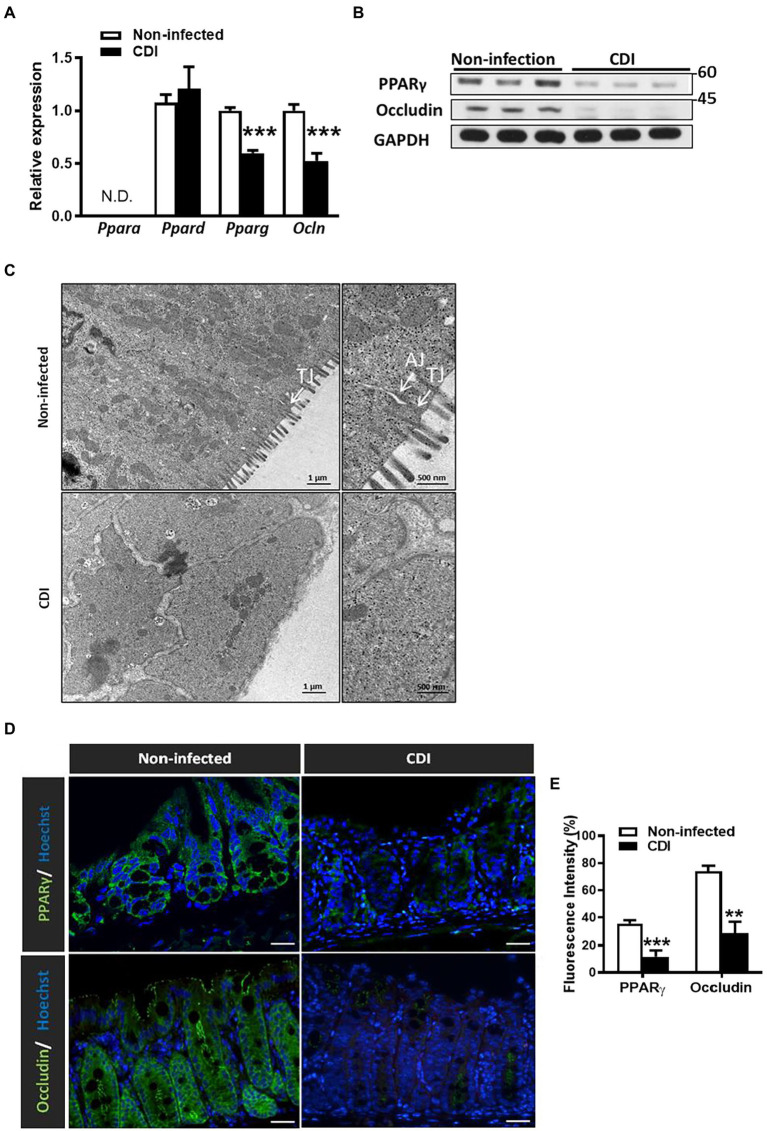
Downregulation of PPAR-γ and occludin in colonic tissue after CDI in C57BL/6JNarl mice. Seven to eight-week-old C57BL/6JNarl mice were orally inoculated with *Clostridioides difficile* for 48 h. **(A)** Real-time PCR of mRNA expression of PPAR family and occludin in colonic tissue. **(B)** Western blot analysis of PPAR-γ and occludin protein levels in CDI colon tissues. **(C)** Representative images of colonic tissue infected with *C. difficile* from mice *via* a transmission electron microscope. White arrows indicate the position of the tight junction (TJ) and adherens junction (AJ) on the microscopic images. Scale bars stand for 1 μm and 500 nm. **(D)** Immunofluorescent staining of PPAR-γ (green), occludin (green), and Hoechst (blue) in colonic tissue of non-infected and *C. difficile*-infected mice. Scale bars stand for 20 μm. **(E)** The percentage of the cells stained with PPAR-γ and occludin relative to the total number of Hoechst^+^ cells were quantified with TissueQuest Analysis software, respectively. The immunofluorescent staining was quantified by five fields of views. Values are expressed as mean ± SEM (**, *p* < 0.01; ***, *p* < 0.001 relative to control group). All data are representative of three independent experiments.

Since PPAR-γ is highly expressed in colonic tissues and negatively regulates intestinal inflammation, we would like to determine whether this colonic transcriptional factor plays a role in CDI (Dubuquoy et al., 2006). To understand the role of PPAR family in intestinal inflammation associated with CDI, we initially examined transcriptional levels of PPAR family in colonic tissues from mice infected with *C. difficile* strain JIK 8284 (A^+^B^+^), based on our previously established animal model (Hung et al., 2015). Notably, we found that only PPAR-γ mRNA expression decreased after 48 h of CDI in colonic tissues (Figure 1A). Moreover, *C. difficile* induced a decrease in PPAR-γ protein level in colonic tissues after 48 h of infection (Figure 1B). Since we have previously reported the intestinal permeability of *C. difficile*-infected mice is higher than that of WT mice, as measured by dextran (molecular weight: 3 kDa) labeled with fluorescein isothiocyanate (FITC) and lipopolysaccharide levels in the serum, gut epithelial integrity was damaged during *C. difficile* infection. Consistently, the mRNA and protein expression of occludin, a tight junction protein, decreased in the colonic tissues of mice with CDI compared to that in non-infected mice (Figures 1A,B). Further, electron micrographs revealed a loss of tight junction providing contact between cells and a larger cell gap in the colon after 48 h of *C. difficil*e challenge (Figure 1C). These findings suggested that the impairment of intestinal epithelial barriers persists in the mice with CDI and is correlated with PPAR-γ expression. To further investigate the changes of PPAR-γ and occludin expression in the mice with CDI, we performed immunofluorescent staining to show their distribution in *C. difficile*-infected colonic tissues and found that PPAR-γ was mainly expressed in the ECs of colonic tissues, while occludin was expressed at the lateral side of the adjacent ECs (Figure 1D). Notably, immunofluorescent analysis showed decreased expression of PPAR-γ and occludin (Figures 1D,E). The decline of PPAR-γ protein and mRNA levels was associated with decreased occludin, indicating that PPAR-γ might contribute to the colonic epithelial integrity in *C. difficile*-infected mice.

### Toxigenic *Clostridioides difficile* decreases peroxisome proliferator-activated receptor-γ and tight junction proteins

**Figure 2:**
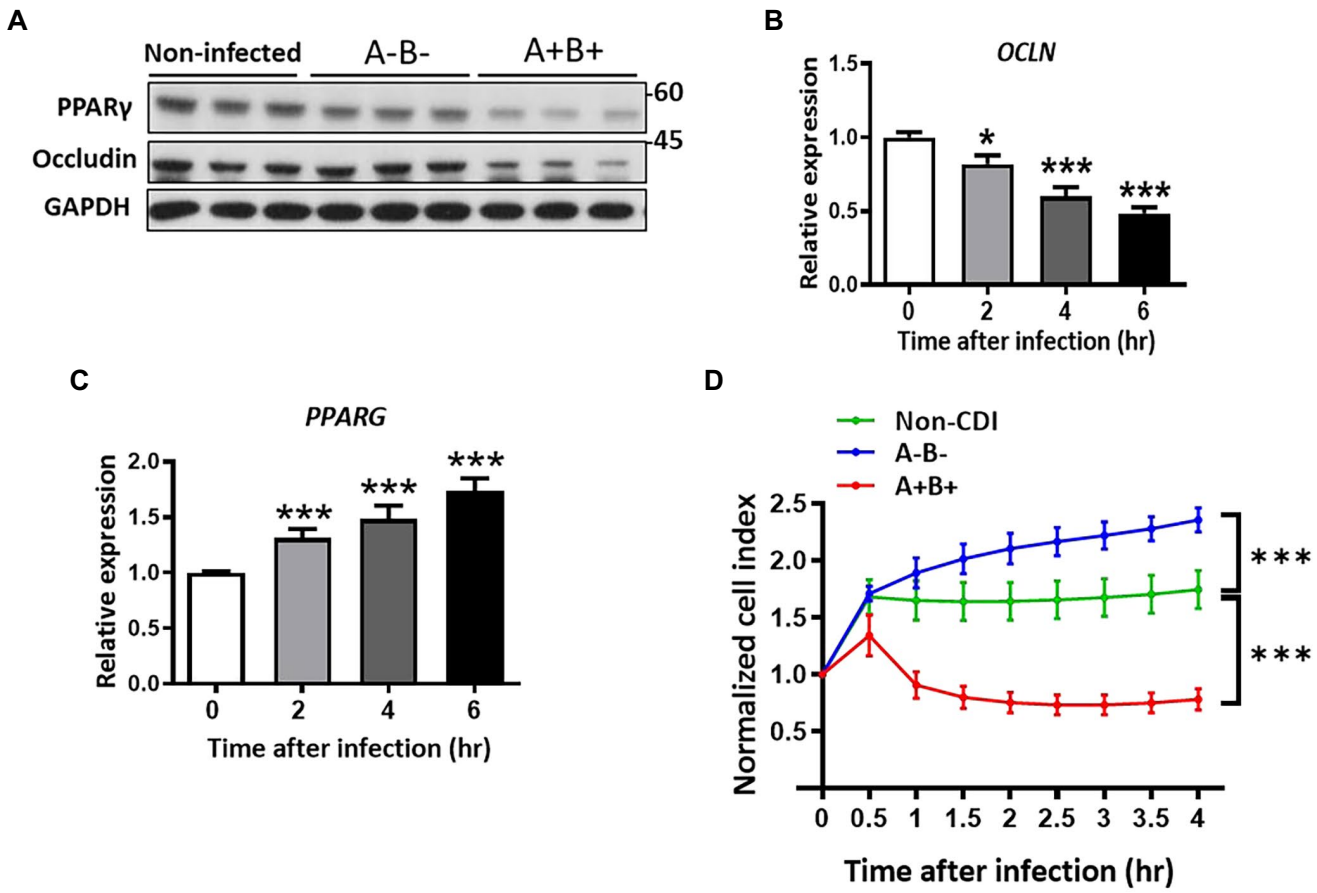
Downregulation of PPAR-γ and occludin associated with loss of cell integrity in *C. difficile*-infected HT-29 cells. Expression of occludin was changed in control and *C. difficile* JIK 8284 (wild type) and isogenic toxin mutant strain DLL 3121 (A^−^B^−^)-infected HT-29 cells. **(A)** Western blot of PPAR-γ and occludin in HT-29 after 4 h postinfection. **(B)** Real-time PCR of occludin expression at the indicated time points. **(C)** Real-time PCR of PPAR-γ expression at the indicated time points. **(D)** Dynamic impedance-based monitoring of CDI-induced epithelial barrier dysfunction in HT-29 cells, which were infected with or without *C. difficile*. All values are normalized to the value of untreated cells and expressed as mean ± SEM (*, *p* < 0.05; **, *p* < 0.01; ***, *p* < 0.001 relative to control group). All data are representative of three independent experiments.

*Clostridioides difficile* produces two exotoxins, toxins A and B, which are recognized as the virulent factors that influence the distribution of tight junction proteins, occludin and ZO-1 (Kuehne et al., 2010; Buccigrossi et al., 2019; Han et al., 2019). Here, we attempted to study the relationship of PPAR-γ and occludin that affected by these toxins. HT-29 epithelial cells were infected with toxigenic strain, JIK 8284 (A+B+),and the isogenic mutant, DLL3121 (A−B−), respectively, we found that the expression of occludin decreased in toxigenic strain-infected cells in a time-dependent manner (Figure 2B), but not in cells infected by the isogenic mutant (Figure 2A). Importantly, the protein level of PPAR-γ and occludin decreased (Figure 2A), whereas the transcriptional level of PPAR-γ increased in cells infected with the toxigenic strain (Figure 2C). These results suggested that the downregulation of both PPAR-γ and occludin protein levels was related to clostridial toxins. Moreover, we examined the temporal effect of toxin-induced epithelial barrier disruption with dynamic monitoring by the impedance-based xCELLigence system (real-time cell analyzer). Epithelial resistance analysis revealed a disrupted epithelial barrier in the presence of clostridial toxins, whereas non-infected cells or cells infected with the isogenic non-toxigenic mutant remained intact (Figure 2D), further supporting the notion that toxigenic *C. difficile*-induced impairment of intestinal epithelial barriers can be attributable to toxin production.

### Hypomorphic peroxisome proliferator-activated receptor-γ mice exhibit severe colitis due to *Clostridioides difficile* infection

**Figure 3:**
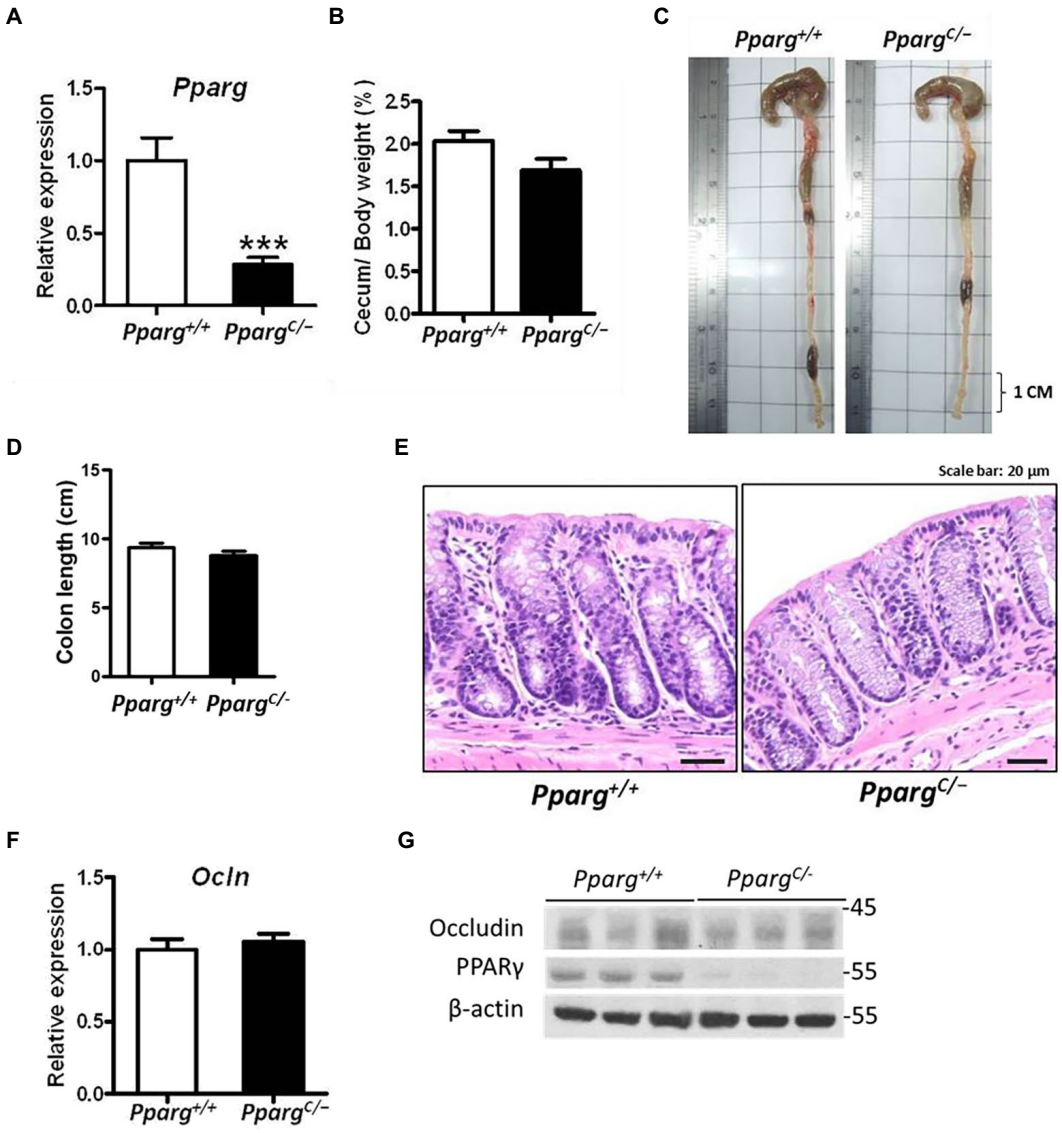
Differences in colonic physiology between wild-type (*Pparg^+/+^*) and PPAR-γ deficient (*Pparg^C/−^*) mice. **(A)** Real-time PCR of PPAR-γ expression in colonic tissue. **(B)** Change of cecum weight divided by body weight. **(C)** Gross view of the colon. **(D)** Quantification of colon length of *Pparg^+/+^* and *Pparg^C/−^* mice without CDI. **(E)** Normal histological pattern and staining for hematoxylin and eosin (H&E) of paraffin-embedded colon tissue prepared from *Pparg^+/+^* and *Pparg^C/−^* mice. **(F)** Real-time PCR of occludin expression in colonic tissue. **(G)** Western blot analysis of PPAR-γ and occludin expression in colonic tissue of *Pparg^+/+^* and *Pparg^C/−^* mice. Values are expressed as mean ± SEM (***, *p* < 0.001 relatives to control group). All data are representative of three independent experiments.

**Figure 4:**
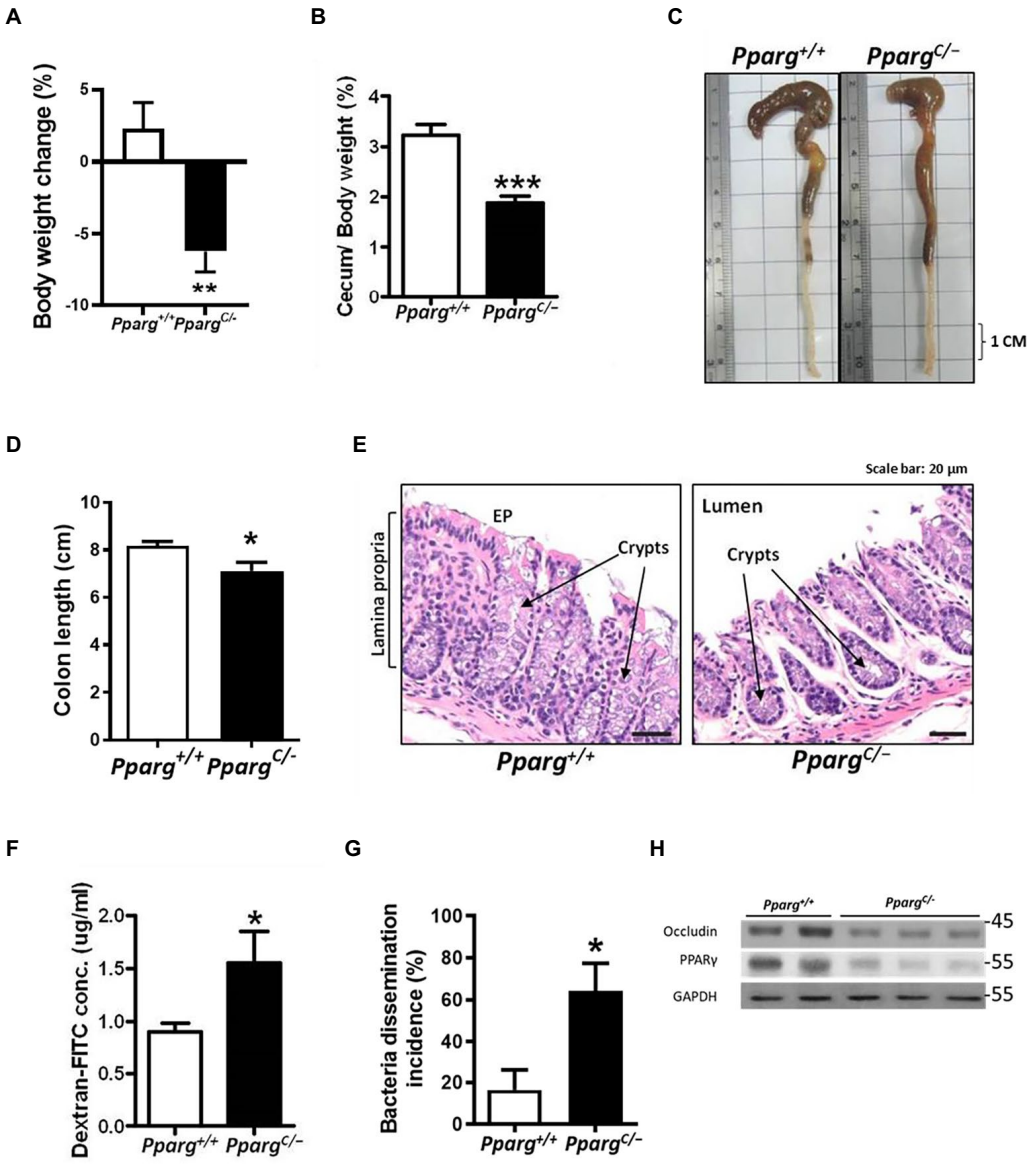
Comparison of pathologic severity and intestinal permeability during CDI between wild-type (*Pparg^+/+^*) mice and PPAR-γ deficient (*Pparg^C/−^*) mice. *C. difficile* were orally gavage to C57BL/6JNarl mice for 48 h. **(A)** Change in body weight after *C. difficile* challenge. **(B)** Change of cecum weight divided by body weight. **(C)** Gross view of the colon. **(D)** Quantification of colon length of *Pparg^+/+^* and *Pparg^c/−^* mice after CDI. **(E)** Histological changes of colon tisues were examined by H&E stain from *Pparg^+/+^* and *Pparg^C/−^* mice. **(F)** Translocation of dextran-FITC in mice compared with wild-type mice (*N* = 9–14/group). **(G)** Percentage of bacterial dissemination to other organs in PPAR-γ deficient mice compared with wild-type mice. **(H)** Western blot analysis of PPAR-γ and occludin expression in colonic tissue of *Pparg^+/+^* and *Pparg^C/−^* mice after CDI. Values are expressed as mean ± SEM (*, *p* < 0.05; **, *p* < 0.01; ***, *p* < 0.001 relative to control group). All data are representative of three independent experiments.

To elucidate the role of PPAR-γ in regulating intestinal barriers during CDI, we utilized *Pparg^C/−^* mice, hypomorphic PPAR-γ mice, with genetic modification in PPAR-γ expression (Tsai et al., 2004). PPAR-γ expression in colonic tissues from *Pparg^C/−^* mice significantly decreased with 25% of PPAR-γ levels in colonic tissues from *Pparg^+/+^* mice (Figure 3A). No detectable changes were observed in cecum and body weights, colon length, and histology between PPAR-γ-deficient and WT mice (Figures 3B–E), indicating that this genetic deficiency did not affect normal physiology of mice. Consistent with the physiological manifestation, mRNA and protein expression levels of occludin were not significantly different in hypomorphic PPAR-γ and WT mice (Figures 3F,G). However, hypomorphic PPAR-γ mice showed a prominent phenotype of CDI colitis, including body and cecum weight loss, decreased colon length, and markedly damaged colonic epithelia in histologic images (Figures 4A–E). To further confirm that PPAR-γ plays a role in the colonic integrity, we assessed intestinal permeability by assessing the leakage of dextran-FITC into the serum and bacterial dissemination to the liver, spleen, and kidney. Higher concentration of dextran-FITC (Figure 4F) and percentage of bacterial dissemination (Figure 4G) were observed in *Pparg^C/−^* mice than those in *Pparg^+/+^* mice with CDI. Moreover, both PPAR-γ and occludin protein expressions were markedly decreased in *Pparg^C/−^* mice compared with *Pparg^+/+^* mice after CDI (Figure 4H). These findings indicate that PPAR-γ deficiency leads to more severe CDI by promoting the disruption of intestinal integrity.

### The peroxisome proliferator-activated receptor-γ agonist reverses gut integrity dysfunction

**Figure 5:**
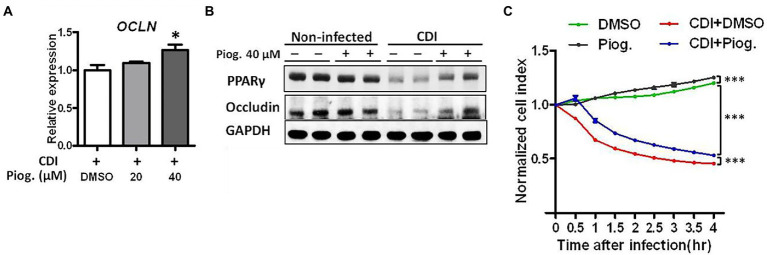
Pioglitazone ameliorated CDI-induced barrier dysfunction in *C. difficile*-infected HT-29 cells. Different dosages of pioglitazone are administered to HT-29 cells 24 h before infection with *C. difficile*. **(A)** The mRNA expression levels of occludin in HT-29 cells were measured by real-time PCR under *C. difficile* infection with or without pioglitazone administration. **(B)** Western blot analysis of occludin and PPAR-γ protein levels in HT-29 cells with or without *C. difficile* infection and with or without pioglitazone administration. **(C)** Using RTCA to measure the cell contact ability altered by *C. difficile* infection with or without pioglitazone administration. HT-29 cells were treated with 20 μM pioglitazone or dimethyl sulfoxide (DMSO) 1 h before CDI. Pioglitazone was dissolved in DMSO, and the same concentration of DMSO was used in the control group. Values are expressed as mean ± SEM (*, *p* < 0.05; ***, *p* < 0.001 relative to control group). All data are representative of three independent experiments.

To investigate the possibility that PPAR-γ activation may contribute to the maintenance of intestinal barrier, we utilized an *in vitro* system to evaluate whether PPAR-γ activation by the PPAR-γ agonist, pioglitazone, reverses CDI-driven tight junction breakage. According to an earlier report, pioglitazone was used to activate PPAR-γ by promoting mitochondrial biogenesis (Ghosh et al., 2007). As expected, the administration of pioglitazone increased the mRNA expression of occludin during CDI. The expression of occludin increased with pioglitazone and was significantly higher at 40 μM pioglitazone compared to DMSO (Figure 5A). We also tested whether occludin were reversed by pioglitazone treatment through PPAR-γ activation. Western blotting analysis clearly showed both PPAR-γ and occludin protein levels were decreased during CDI, whereas both were reversed by administering 40 μM pioglitazone (Figure 5B), demonstrating that activation of PPAR-γ might participate in the regulation of occludin protein expression. Further examination of epithelial integrity using an impedance-based xCELLigence system showed that CDI-induced barrier dysfunction was reversed following pioglitazone administration in HT-29 cells compared to that in *C. difficile-*infected cells treated with DMSO solvent (Figure 5C), highlighting the causal link between PPAR-γ and tight junctions in the gut.

### The peroxisome proliferator-activated receptor-γ agonist ameliorates *Clostridioides difficile* infection severity

**Figure 6:**
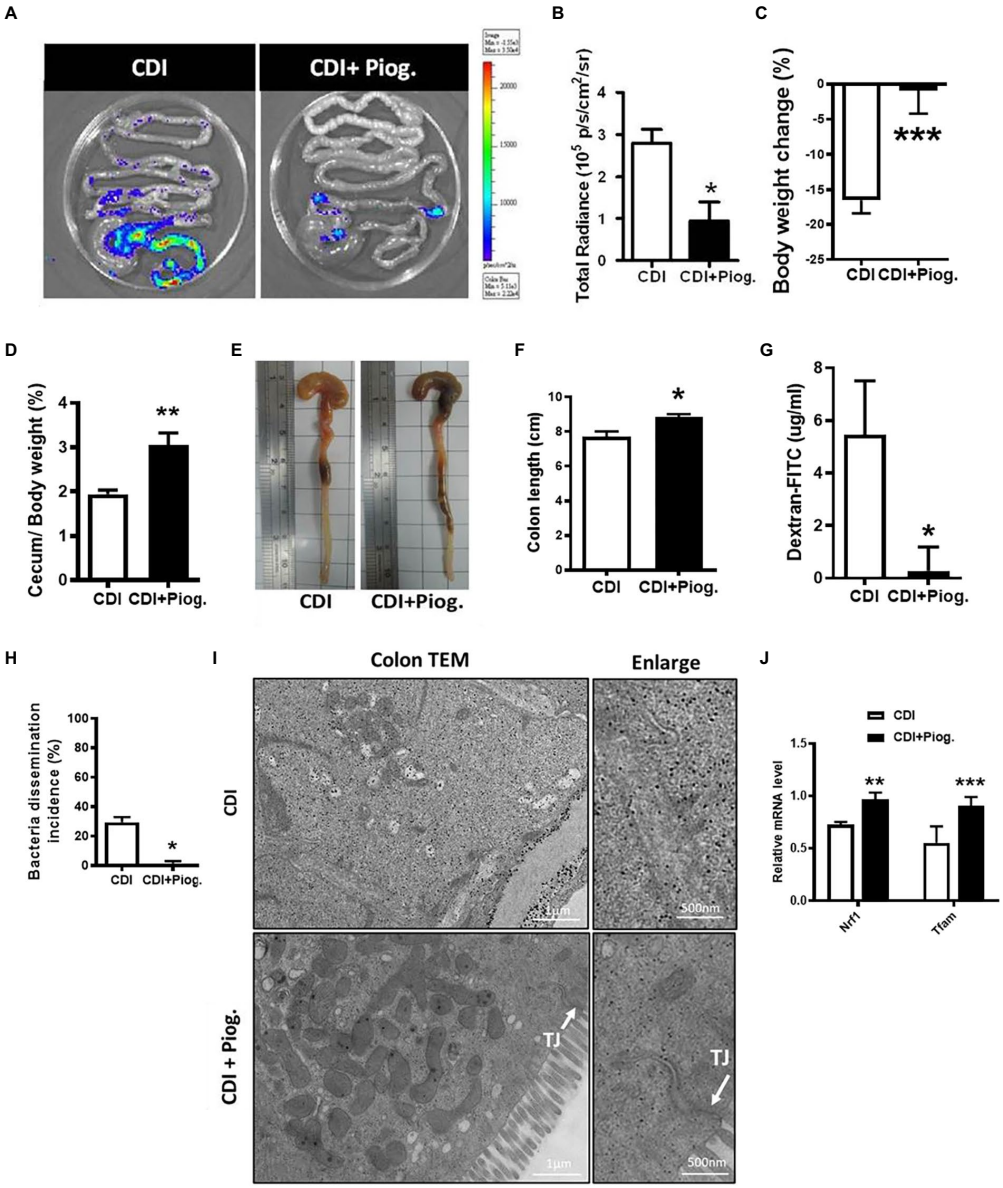
Pioglitazone attenuated *C. difficile*-induced inflammation and barrier dysfunction in NF-κB-RE-Luc reporter mice. NF-κB-RE-Luc reporter mice were either left untreated or treated with pioglitazone orally for 5 days and then challenged with *C. difficile* for 48 h. **(A)** Activation of NF-κB in the cecum and colon was monitored by using *in vivo* bioluminescence imaging. **(B)** Quantification of luminescence levels in the gut from CDI mice with or without pioglitazone treatment. **(C–F)** Change in body weight, change of cecum weight divided by body weight, gross view of cecum and colon, and colon length were assessed 2 days after infection. **(G)** Translocation of dextran-FITC from CDI mice with or without pioglitazone treatment. **(H)** Percentage of bacterial dissemination to other organs in mice with or without pioglitazone. **(I)** Transmission electron micrograph of colon tissues from CDI mice with or without administration of pioglitazone. White arrows indicate the position of the tight junction on the microscopic images of CDI with pioglitazone treatment. Scale bars stand for 1 μm and 500 nm. **(J)** PPAR-γ activation triggered by pioglitazone was monitored by PPAR-γ downstream genes. RT-qPCR analysis of the expression of indicated genes in colon tissue of *C. difficile* infection with or without pioglitazone treatment on day 2 after infection. Values are expressed as mean ± SEM (*, *p* < 0.05; **, *p* < 0.01; ***, *p* < 0.001 relative to control group). All data are representative of five independent experiments.

After we demonstrated that activation of PPAR-γ regulates occludin expression during CDI, we would like to assess whether the activation of PPAR-γ repairs the gut barrier during CDI *in vivo*. Since Nuclear factor-κB (NF-κB) serves as transcription factor that regulates genes involved in complex and diverse processes in immunity and inflammation response (Liu et al., 2017), we utilized FVB/JNarl background NF-κB-RE-Luc reporter mice to observe CDI-induced colon inflammation. We had used this NF-κB-RE-Luc reporter mice to elucidate the disease progression of CDI in previous studies (Hung et al., 2015; Lee et al., 2017). Pioglitazone has been used in mice with a wide range of doses for studying PPAR-γ-associated disease (Niho et al., 2003; Bogacka et al., 2005; Kawasaki et al., 2005; Masciopinto et al., 2012; Garcia-Ruiz et al., 2013). Based on previous animal studies, we treated mice with 70 mg/kg pioglitazone for 5 days before CDI. Changes in body weight and loss of cecum weight induced by CDI were improved in the group which was treated with pioglitazone (data not shown). The luminescent signal which represented NF-κB activation in the intestinal tract was reversed in CDI mice treated with pioglitazone compared to non-treated mice (Figures 6A,B). Moreover, mice treated with pioglitazone showed improved body and cecum weight loss, and shortened colon length (Figures 6C–F). In addition, pioglitazone administration further decreases the leakage of dextran-FITC into serum. Similarly, almost no bacterial dissemination was observed in *C. difficile*-infected mice treated with pioglitazone compared to those non-treated mice (Figures 6G,H). Moreover, TEM images also clearly showed a reduced gap width between cells and tight junction in pioglitazone treated mice, compared with CDI mice (Figure 6I). To confirm PPAR-γ was activated after administration of pioglitazone, the expressions of PPAR-γ downstream genes, including nuclear respiratory factor 1 (NRF1) and mitochondrial transcription factor A (mtTFA), were measured. Results from real-time PCR analysis showed that the expressions of NRF1 and mtTFAM in colon tissue of CDI mice were reduced to around 50% expression levels of non-infected mice, whereas both genes were markedly reversed by pioglitazone treatment, compared to non-infected mice (Figure 6J). These findings illustrated that PPAR-γ activation by pioglitazone alleviated symptoms through not only the anti-inflammatory effect but also the improvement of intestinal integrity. Collectively, these findings suggest that PPAR-γ can regulate tight junctions, which exert a profound influence on CDI severity.

## Discussion

*Clostridioides difficile* infection is a major nosocomial infection with a high recurrence rate worldwide (Kassam et al., 2016). To date, different strains of *C. difficile* have caused several outbreaks and lead to ~29,000 deaths each year in the United States (Lessa et al., 2015). Metronidazole and vancomycin are the primary drugs used to treat CDI (Crobach et al., 2016). However, the use of these drugs may facilitate vancomycin-resistant enterococci colonization (Gerding, 1997) and high recurrence rate (Gerding et al., 2008; DuPont, 2011). The use of new drugs, such as ramoplanin, rifamycin, nitazoxanide, and fidaxomicin, or conventional drugs, such as fusidic acid, teicoplanin, and rifampin, to treat CDI is either still under investigation or not currently available in Taiwan (Hedge et al., 2008). In our study, we found that *C. difficile* downregulated PPAR-γ expression in colonic epithelial cells associated with decreased expression of tight junction proteins and disruption of gut integrity, which were restored by the administration of a PPAR-γ agonist (Figures 5A–C). The PPAR-γ agonist, pioglitazone, was initially used as an anti-diabetes medication; however, it has recently been used for treating inflammatory bowel disease, such as UC (Lewis et al., 2001, 2008). Our results suggest that the PPAR-γ agonist might serve as an alternative or combination antimicrobial regimen for the treatment of CDI, based on *in vitro* or *in vivo* evidence.

Our study showed that gut epithelium integrity as a natural barrier is important for protection against *C. difficile* infection. *Clostridioides difficile* toxin A has been found to induce the disruption of epithelial integrity, including severe hemorrhagic and inflammatory fluid secretion at 6–8 h postinfection in rabbit ileal segments (Lima et al., 2008). The recombinant repetitive domain of *C. difficile* toxin B obtained from two different strains, rec-TcdB3 (10463) and rec-TcdB3 (8864), on model intestinal epithelial cells caused intestinal epithelial cell damages, including decreased transepithelial electrical resistance, and induced the translocation of zonula occludens-1 from tight junction proteins (Zemljic et al., 2010). The gut epithelial barrier might serve as an important therapeutic intervention target for CDI. Indeed, our results demonstrated that both mRNA and protein expression of occludin, a tight junction protein, was decreased during CDI *in vitro* (Figures 1A,B) and that gut barrier permeability was increased after CDI, as evidenced by the increased levels of FITC-labeled dextran (Figure 6F) *in vivo*.

The role of PPAR-γ in the regulation of intestinal barrier during CDI was explored for the first time in the present study, in contrast to investigation of the anti-inflammatory effects of PPAR-γ on the gut in previous studies. PPAR-γ ligands have been shown to attenuate inflammatory cytokine production (e.g., IL-8 and tumor necrosis factor-α; Krentz and Bailey, 2005; Wang et al., 2017), inflammatory cell proliferation, and expression of selected adhesion molecules. Furthermore, treatment with PPAR-γ ligands reduces colonic inflammation in several murine models of colitis (Su et al., 1999; Dubuquoy et al., 2006). Viladomiu et al. found that the colonic expression of IL-17 was upregulated, while that of IL-10 was downregulated in T cell-specific PPAR-γ null mice. Moreover, the loss of PPAR-γ in T cells increased disease severity and colonic inflammation in CDI (Viladomiu et al., 2012). Notably, PPAR hypomorphic mice used in this study not only have an impact on tight junction expression levels but also have decreased T-helper 17 cell counts, as demonstrated previously (Liu et al., 2016). Our study demonstrated that the expression of colonic PPAR-γ in CDI, independent of the anti-inflammatory pathway, had a role in maintaining the integrity of the gut barrier, as evidenced by occludin expression, xCELLigence system, bacterial dissemination, and dextran-FITC translocation analysis.

Nevertheless, there are some drawbacks to our study. First, we only investigated the role of PPAR-γ in colon barrier instead of its anti-inflammatory effect, which has been investigated in previous studies (Yang et al., 2021). Inflammation in the gut might have a role in colonic barrier protection, but this needs further investigation. Second, although the PPAR-γ agonist has been used clinically for many years, we did not know whether it solely affects the intestinal epithelial cells; this warrants further study before the PPAR-γ agonist can be used to treat CDI clinically. Third, metronidazole and vancomycin were the drug choices for treating CDI, further combination treatment of PPAR-γ agonist with metronidazole and vancomycin need to be explored. Nevertheless, to the best of our knowledge, this is the first study to reveal the effect of the PPAR-γ agonist on colonic barrier integrity and CDI severity.

Our findings demonstrated that *C. difficile*-induced PPAR-γ downregulation in colonic epithelial cells, which associated with the decreased tight junction proteins and disruption of gut integrity, can be reversed by administering the PPAR-γ agonist. Our results indicate that activation of PPAR-γ rescues the disruption of tight junctions, and provides a new therapeutic strategy.

## Data availability statement

The original contributions presented in the study are included in the article/supplementary material, further inquiries can be directed to the corresponding authors.

## Ethics statement

The animal study was reviewed and approved by National Laboratory Animal Center.

## Author contributions

T-CW, Y-ST, P-JT, and W-CK designed the experiments. Y-HL, T-CW, and B-YT performed the experiments and analyzed the data. Y-PH, H-JL, and Y-ST contributed animals, reagents, materials, and analysis tools. T-CW prepared the original draft. Y-HL edited the manuscript. Y-ST, W-CK, and P-JT revised and supervised the study. All authors contributed to the article and approved the submitted version.

## Funding

This work is supported by research grants from the Ministry of Science and Technology (MOST; 108-2320-B-006 -043 -MY3; 109-2314-B-006-089-MY3; 110-2314-B-675-001 and 111-2320-B-006 -046 -MY3).

## Conflict of interest

The authors declare that the research was conducted in the absence of any commercial or financial relationships that could be construed as a potential conflict of interest.

## Publisher’s note

All claims expressed in this article are solely those of the authors and do not necessarily represent those of their affiliated organizations, or those of the publisher, the editors and the reviewers. Any product that may be evaluated in this article, or claim that may be made by its manufacturer, is not guaranteed or endorsed by the publisher.

## References

[ref1] Bogacka I. Xie H. Bray G. A. Smith S. R. (2005). Pioglitazone induces mitochondrial biogenesis in human subcutaneous adipose tissue in vivo. Diabetes 54, 1392–1399. doi: 10.2337/diabetes.54.5.1392, PMID: 15855325

[ref2] Buccigrossi V. Lo Vecchio A. Marano A. Guarino A. (2019). Differential effects of *Clostridium difficile* toxins on ion secretion and cell integrity in human intestinal cells. Pediatr. Res. 85, 1048–1054. doi: 10.1038/s41390-019-0365-0, PMID: 30851723

[ref3] Chen S. Sun C. Wang H. Wang J. (2015). The role of rho GTPases in toxicity of *Clostridium difficile* toxins. Toxins. 7, 5254–5267. doi: 10.3390/toxins7124874, PMID: 26633511 PMC4690124

[ref4] Chen J. Y. Wu Y. P. Li C. Y. Jheng H. F. Kao L. Z. Yang C. C.. (2021). PPARgamma activation improves the microenvironment of perivascular adipose tissue and attenuates aortic stiffening in obesity. J. Biomed. Sci. 28:22. doi: 10.1186/s12929-021-00720-y, PMID: 33781257 PMC8008548

[ref5] Chiu C. W. Tsai P. J. Lee C. C. Ko W. C. Hung Y. P. (2021). Application of microbiome management in therapy for *Clostridioides difficile* infections: from fecal microbiota transplantation to probiotics to microbiota-preserving antimicrobial agents. Pathogens 10, 649–662. doi: 10.3390/pathogens10060649 34073695 PMC8225043

[ref6] Cho J. M. Pardi D. S. Khanna S. (2020). Update on treatment of *Clostridioides difficile* infection. Mayo Clin. Proc. 95, 758–769. doi: 10.1016/j.mayocp.2019.08.006 32247350

[ref7] Colleen R. Kelly A. K. Staley C. Sadowsky M. J. Abd M. Alani M.. (2016). Effect of fecal microbiota transplantation on recurrence in multiply recurrent *Clostridium difficile* infection. Ann. Intern. Med. 165, 609–616. doi: 10.7326/M16-0271 27547925 PMC5909820

[ref8] Cornely O. A. Miller M. A. Louie T. J. Crook D. W. Gorbach S. L. (2012). Treatment of first recurrence of *Clostridium difficile* infection: fidaxomicin versus vancomycin. Clin. Infect. Dis. 55, S154–S161. doi: 10.1093/cid/cis462, PMID: 22752865 PMC3388030

[ref9] Cowardin C. A. Jackman B. M. Noor Z. Burgess S. L. Feig A. L. Petri W. A. Jr. (2016). Glucosylation drives the innate inflammatory response to *Clostridium difficile* toxin a. Infect. Immun. 84, 2317–2323. doi: 10.1128/IAI.00327-16, PMID: 27271747 PMC4962640

[ref10] Crobach M. J. Planche T. Eckert C. Barbut F. Terveer E. M. Dekkers O. M.. (2016). European Society of Clinical Microbiology and Infectious Diseases: update of the diagnostic guidance document for *Clostridium difficile* infection. Clin. Microbiol. Infect. 22, S63–S81. doi: 10.1016/j.cmi.2016.03.010, PMID: 27460910

[ref11] Di Bella S. Ascenzi P. Siarakas S. Petrosillo N. di Masi A. (2016). *Clostridium difficile* toxins a and B: insights into pathogenic properties and Extraintestinal effects. Toxins 8, 134–158. doi: 10.3390/toxins8050134, PMID: 27153087 PMC4885049

[ref12] Dilnessa T. Getaneh A. Hailu W. Moges F. Gelaw B. (2022). Prevalence and antimicrobial resistance pattern of *Clostridium difficile* among hospitalized diarrheal patients: a systematic review and meta-analysis. PLoS One 17:e0262597. doi: 10.1371/journal.pone.0262597, PMID: 35025959 PMC8758073

[ref13] Dou X. Xiao J. Jin Z. Zheng P. (2015). Peroxisome proliferator-activated receptor-gamma is downregulated in ulcerative colitis and is involved in experimental colitis-associated neoplasia. Oncol. Lett. 10, 1259–1266. doi: 10.3892/ol.2015.3397, PMID: 26622660 PMC4533289

[ref14] Dubuquoy L. Dharancy S. Nutten S. Pettersson S. Auwerx J. Desreumaux P. (2002). Role of peroxisome proliferator-activated receptor gamma and retinoid X receptor heterodimer in hepatogastroenterological diseases. Lancet 360, 1410–1418. doi: 10.1016/S0140-6736(02)11395-X, PMID: 12424006

[ref15] Dubuquoy L. Jansson E. A. Deeb S. Rakotobe S. Karoui M. Colombel J. F.. (2003). Impaired expression of peroxisome proliferator-activated receptor gamma in ulcerative colitis. Gastroenterology 124, 1265–1276. doi: 10.1016/S0016-5085(03)00271-3, PMID: 12730867

[ref16] Dubuquoy L. Rousseaux C. Thuru X. Peyrin-Biroulet L. Romano O. Chavatte P.. (2006). PPARgamma as a new therapeutic target in inflammatory bowel diseases. Gut 55, 1341–1349. doi: 10.1136/gut.2006.093484, PMID: 16905700 PMC1860011

[ref17] DuPont H. L. (2011). The search for effective treatment of *Clostridium difficile* infection. N. Engl. J. Med. 364, 473–475. doi: 10.1056/NEJMe1013236, PMID: 21288079

[ref18] Garcia-Ruiz I. Solis-Munoz P. Fernandez-Moreira D. Munoz-Yague T. Solis-Herruzo J. A. (2013). Pioglitazone leads to an inactivation and disassembly of complex I of the mitochondrial respiratory chain. BMC Biol. 11:88. doi: 10.1186/1741-7007-11-88, PMID: 23915000 PMC3751493

[ref19] Garey K. W. Jiang Z. D. Ghantoji S. Tam V. H. Arora V. Dupont H. L. (2010). A common polymorphism in the interleukin-8 gene promoter is associated with an increased risk for recurrent *Clostridium difficile* infection. Clin. Infect. Dis. 51, 1406–1410. doi: 10.1086/657398, PMID: 21058913

[ref20] Gerding D. N. (1997). Is there a relationship between vancomycin-resistant enterococcal infection and *Clostridium difficile* infection? Clin. Infect. Dis. 25, S206–S210. doi: 10.1086/516247 9310680

[ref21] Gerding D. N. Muto C. A. Owens R. C. Jr. (2008). Treatment of *Clostridium difficile* infection. Clin. Infect. Dis. 46, S32–S42. doi: 10.1086/521860 18177219

[ref22] Gerhard R. Queisser S. Tatge H. Meyer G. Dittrich-Breiholz O. Kracht M.. (2011). Down-regulation of interleukin-16 in human mast cells HMC-1 by *Clostridium difficile* toxins A and B. Naunyn. Schmiedeberg's Arch. Pharmacol. 383, 285–295. doi: 10.1007/s00210-010-0592-8, PMID: 21267712

[ref23] Ghosh S. Patel N. Rahn D. McAllister J. Sadeghi S. Horwitz G.. (2007). The thiazolidinedione pioglitazone alters mitochondrial function in human neuron-like cells. Mol. Pharmacol. 71, 1695–1702. doi: 10.1124/mol.106.033845, PMID: 17387142

[ref24] Gil F. Calderon I. L. Fuentes J. A. Paredes-Sabja D. (2018). *Clostridioides (clostridium) difficile* infection: current and alternative therapeutic strategies. Future Microbiol. 13, 469–482. doi: 10.2217/fmb-2017-0203 29464969

[ref25] Han S. H. Yi J. Kim J. H. Lee S. Moon H. W. (2019). Composition of gut microbiota in patients with toxigenic *Clostridioides (clostridium) difficile*: comparison between subgroups according to clinical criteria and toxin gene load. PLoS One 14:e0212626. doi: 10.1371/journal.pone.0212626, PMID: 30785932 PMC6382146

[ref26] Hedge D. D. Strain J. D. Heins J. R. Farver D. K. (2008). New advances in the treatment of *Clostridium difficile* infection (CDI). Ther. Clin. Risk Manag. 4, 949–964. 19209277 10.2147/tcrm.s3145 PMC2621401

[ref27] Huang W. Eum S. Y. Andras I. E. Hennig B. Toborek M. (2009). PPARalpha and PPARgamma attenuate HIV-induced dysregulation of tight junction proteins by modulations of matrix metalloproteinase and proteasome activities. FASEB J. 23, 1596–1606. doi: 10.1096/fj.08-121624, PMID: 19141539 PMC2669424

[ref28] Hung Y. P. Ko W. C. Chou P. H. Chen Y. H. Lin H. J. Liu Y. H.. (2015). Proton-pump inhibitor exposure aggravates *Clostridium difficile*-associated colitis: evidence from a mouse model. J. Infect. Dis. 212, 654–663. doi: 10.1093/infdis/jiv184, PMID: 25805751

[ref29] Kassam Z. Cribb Fabersunne C. Smith M. B. Alm E. J. Kaplan G. G. Nguyen G. C.. (2016). *Clostridium difficile* associated risk of death score (CARDS): a novel severity score to predict mortality among hospitalised patients with *C. difficile* infection. Aliment. Pharmacol. Ther. 43, 725–733. doi: 10.1111/apt.13546, PMID: 26849527 PMC5094350

[ref30] Kawasaki F. Matsuda M. Kanda Y. Inoue H. Kaku K. (2005). Structural and functional analysis of pancreatic islets preserved by pioglitazone in db/db mice. Am. J. Physiol. Endocrinol. Metab. 288, E510–E518. doi: 10.1152/ajpendo.00128.2004, PMID: 15522998

[ref31] Kelly C. P. LaMont J. T. (2008). *Clostridium difficile*–more difficult than ever. N. Engl. J. Med. 359, 1932–1940. doi: 10.1056/NEJMra0707500, PMID: 18971494

[ref32] Khanna S. Pardi D. S. Kelly C. R. Kraft C. S. Dhere T. Henn M. R.. (2016). A novel microbiome therapeutic increases gut microbial diversity and prevents recurrent *Clostridium difficile* infection. J. Infect. Dis. 214, 173–181. doi: 10.1093/infdis/jiv766, PMID: 26908752

[ref33] Krentz A. J. Bailey C. J. (2005). Oral antidiabetic agents: current role in type 2 diabetes mellitus. Drugs 65, 385–411. doi: 10.2165/00003495-200565030-00005 15669880

[ref34] Kuehne S. A. Cartman S. T. Heap J. T. Kelly M. L. Cockayne A. Minton N. P. (2010). The role of toxin a and toxin B in *Clostridium difficile* infection. Nature 467, 711–713. doi: 10.1038/nature09397 20844489

[ref35] Lai Y. H. Tsai B. Y. Hsu C. Y. Chen Y. H. Chou P. H. Chen Y. L.. (2021). The role of toll-like Receptor-2 in Clostridioides difficile infection: evidence from a mouse model and clinical patients. Front. Immunol. 12:691039. doi: 10.3389/fimmu.2021.691039, PMID: 34322122 PMC8313301

[ref36] Lee W. T. Wu Y. N. Chen Y. H. Wu S. R. Shih T. M. Li T. J.. (2017). Octahedron iron oxide nanocrystals prohibited *Clostridium difficile* spore germination and attenuated local and systemic inflammation. Sci. Rep. 7:8124. doi: 10.1038/s41598-017-08387-y, PMID: 28811642 PMC5558001

[ref37] Lessa F. C. Winston L. G. McDonald L. C. (2015). Emerging infections program CdST. Burden of *Clostridium difficile* infection in the United States. N. Engl. J. Med. 372, 2369–2370. doi: 10.1056/NEJMc1505190, PMID: 26061850 PMC10880113

[ref38] Lewis J. D. Lichtenstein G. R. Deren J. J. Sands B. E. Hanauer S. B. Katz J. A.. (2008). Rosiglitazone for active ulcerative colitis: a randomized placebo-controlled trial. Gastroenterology 134, 688–695. doi: 10.1053/j.gastro.2007.12.012, PMID: 18325386 PMC2276587

[ref39] Lewis J. D. Lichtenstein G. R. Stein R. B. Deren J. J. Judge T. A. Fogt F.. (2001). An open-label trial of the PPAR-gamma ligand rosiglitazone for active ulcerative colitis. Am. J. Gastroenterol. 96, 3323–3328. 11774944 10.1111/j.1572-0241.2001.05333.x

[ref40] Li H. Singh S. Potula R. Persidsky Y. Kanmogne G. D. (2014). Dysregulation of claudin-5 in HIV-induced interstitial pneumonitis and lung vascular injury. Protective role of peroxisome proliferator-activated receptor-gamma. Am. J. Respir. Crit. Care Med. 190, 85–97. doi: 10.1164/rccm.201106-1151OC, PMID: 22345580 PMC4226025

[ref41] Lima A. A. Nascimento N. R. Fang G. D. Yotseff P. Toyama M. H. Guerrant R. L.. (2008). Role of phospholipase A2 and tyrosine kinase in *Clostridium difficile* toxin a-induced disruption of epithelial integrity, histologic inflammatory damage and intestinal secretion. J. Appl. Toxicol. 28, 849–857. doi: 10.1002/jat.1348, PMID: 18381687

[ref42] Liu Y. H. Chang Y. C. Chen L. K. Su P. A. Ko W. C. Tsai Y. S.. (2018). The ATP-P2X7 signaling Axis is an essential sentinel for intracellular *Clostridium difficile* pathogen-induced Inflammasome activation. Front. Cell. Infect. Microbiol. 8:84. doi: 10.3389/fcimb.2018.00084, PMID: 29616195 PMC5864904

[ref43] Liu Y. H. Tsai Y. S. Lin S. C. Liao N. S. Jan M. S. Liang C. T.. (2016). Quantitative PPARgamma expression affects the balance between tolerance and immunity. Sci. Rep. 6:26646. doi: 10.1038/srep26646, PMID: 27221351 PMC4879582

[ref44] Liu T. Zhang L. Joo D. Sun S. C. (2017). NF-kappaB signaling in inflammation. Signal Transduct. Target. Ther. 2:2. doi: 10.1038/sigtrans.2017.23 PMC5661633 29158945

[ref45] Masciopinto F. Di Pietro N. Corona C. Bomba M. Pipino C. Curcio M.. (2012). Effects of long-term treatment with pioglitazone on cognition and glucose metabolism of PS1-KI, 3xTg-AD, and wild-type mice. Cell Death Dis. 3:e448. doi: 10.1038/cddis.2012.189, PMID: 23254291 PMC3542623

[ref46] Mileto S. Das A. Lyras D. (2019). Enterotoxic clostridia: *Clostridioides difficile* infections. Microbiol. Spectr. 7, 11–26. doi: 10.1128/microbiolspec.GPP3-0015-2018, PMID: 31124432 PMC11026080

[ref47] Mirza A. Z. Althagafi I. I. Shamshad H. (2019). Role of PPAR receptor in different diseases and their ligands: physiological importance and clinical implications. Eur. J. Med. Chem. 166, 502–513. doi: 10.1016/j.ejmech.2019.01.067, PMID: 30739829

[ref48] Mohapatra S. K. Guri A. J. Climent M. Vives C. Carbo A. Horne W. T.. (2010). Immunoregulatory actions of epithelial cell PPAR gamma at the colonic mucosa of mice with experimental inflammatory bowel disease. PLoS One 5:e10215. doi: 10.1371/journal.pone.0010215, PMID: 20422041 PMC2857885

[ref49] Niho N. Takahashi M. Shoji Y. Takeuchi Y. Matsubara S. Sugimura T.. (2003). Dose-dependent suppression of hyperlipidemia and intestinal polyp formation in min mice by pioglitazone, a PPAR gamma ligand. Cancer Sci. 94, 960–964. doi: 10.1111/j.1349-7006.2003.tb01385.x, PMID: 14611672 PMC11160263

[ref50] Ogasawara N. Kojima T. Go M. Ohkuni T. Koizumi J. Kamekura R.. (2010). PPARgamma agonists upregulate the barrier function of tight junctions *via* a PKC pathway in human nasal epithelial cells. Pharmacol. Res. 61, 489–498. doi: 10.1016/j.phrs.2010.03.002, PMID: 20227502

[ref51] Paredes-Sabja D. Shen A. Sorg J. A. (2014). *Clostridium difficile* spore biology: sporulation, germination, and spore structural proteins. Trends Microbiol. 22, 406–416. doi: 10.1016/j.tim.2014.04.003, PMID: 24814671 PMC4098856

[ref52] Ryan A. Lynch M. Smith S. M. Amu S. Nel H. J. McCoy C. E.. (2011). A role for TLR4 in *Clostridium difficile* infection and the recognition of surface layer proteins. PLoS Pathog. 7:e1002076. doi: 10.1371/journal.ppat.1002076, PMID: 21738466 PMC3128122

[ref53] Shah Y. M. Morimura K. Gonzalez F. J. (2007). Expression of peroxisome proliferator-activated receptor-gamma in macrophage suppresses experimentally induced colitis. Am. J. Physiol. Gastrointest. Liver Physiol. 292, G657–G666. doi: 10.1152/ajpgi.00381.2006, PMID: 17095756 PMC1796914

[ref54] Sholeh M. Krutova M. Forouzesh M. Mironov S. Sadeghifard N. Molaeipour L.. (2020). Antimicrobial resistance in *Clostridioides (clostridium) difficile* derived from humans: a systematic review and meta-analysis. Antimicrob. Resist. Infect. Control 9:158. doi: 10.1186/s13756-020-00815-5, PMID: 32977835 PMC7517813

[ref55] Simeoli R. Mattace Raso G. Pirozzi C. Lama A. Santoro A. Russo R.. (2016). An orally administered butyrate-releasing derivative reduces neutrophil recruitment and inflammation in dextran sulphate sodium-induced murine colitis. Br. J. Pharmacol. 174, 1484–1496. 27684049 10.1111/bph.13637 PMC5429328

[ref56] Simpson M. Frisbee A. Kumar P. Schwan C. Aktories K. Petri W. A. (2022). *Clostridioides difficile* binary toxin is recognized by the toll-like receptor 2/6 heterodimer to induce a nuclear factor-kappaB response. J. Infect. Dis. 225, 1296–1300. doi: 10.1093/infdis/jiaa620, PMID: 33011801 PMC8974845

[ref57] Straus D. S. Glass C. K. (2007). Anti-inflammatory actions of PPAR ligands: new insights on cellular and molecular mechanisms. Trends Immunol. 28, 551–558. doi: 10.1016/j.it.2007.09.003, PMID: 17981503

[ref58] Su W. Bush C. R. Necela B. M. Calcagno S. R. Murray N. R. Fields A. P.. (2007). Differential expression, distribution, and function of PPAR-gamma in the proximal and distal colon. Physiol. Genomics 30, 342–353. doi: 10.1152/physiolgenomics.00042.2007, PMID: 17519361

[ref59] Su C. G. Wen X. Bailey S. T. Jiang W. Rangwala S. M. Keilbaugh S. A.. (1999). A novel therapy for colitis utilizing PPAR-gamma ligands to inhibit the epithelial inflammatory response. J. Clin. Invest. 104, 383–389. doi: 10.1172/JCI7145, PMID: 10449430 PMC408529

[ref60] Takaki K. Mitsuyama K. Tsuruta O. Toyonaga A. Sata M. (2006). Attenuation of experimental colonic injury by thiazolidinedione agents. Inflamm. Res. 55, 10–15. doi: 10.1007/s00011-005-0002-8, PMID: 16328104

[ref61] Tsai Y. S. Kim H. J. Takahashi N. Kim H. S. Hagaman J. R. Kim J. K.. (2004). Hypertension and abnormal fat distribution but not insulin resistance in mice with P465L PPARgamma. J. Clin. Invest. 114, 240–249. doi: 10.1172/JCI200420964 15254591 PMC449746

[ref62] Tsigrelis C. (2020). Recurrent *Clostridioides difficile* infection: recognition, management, prevention. Cleve. Clin. J. Med. 87, 347–359. doi: 10.3949/ccjm.87gr.20001, PMID: 32487555

[ref63] Varley C. L. Garthwaite M. A. Cross W. Hinley J. Trejdosiewicz L. K. Southgate J. (2006). PPARgamma-regulated tight junction development during human urothelial cytodifferentiation. J. Cell. Physiol. 208, 407–417. doi: 10.1002/jcp.20676, PMID: 16688762 PMC1522040

[ref64] Viladomiu M. Hontecillas R. Pedragosa M. Carbo A. Hoops S. Michalak P.. (2012). Modeling the role of peroxisome proliferator-activated receptor gamma and microRNA-146 in mucosal immune responses to *Clostridium difficile*. PLoS One 7:e47525. doi: 10.1371/journal.pone.0047525, PMID: 23071818 PMC3469550

[ref65] Wang F. Liu Y. Bi Z. (2017). Pioglitazone inhibits growth of human retinoblastoma cells *via* regulation of NF-kappaB inflammation signals. J. Recept. Signal Transduct. Res. 37, 94–99. doi: 10.3109/10799893.2016.1171341, PMID: 27133446

[ref66] Weingarden A. González A. Vázquez-Baeza Y. Weiss S. Humphry G. Berg-Lyons D.. (2015). Dynamic changes in short- and long-term bacterial composition following fecal microbiota transplantation for recurrent *Clostridium difficile* infection. Microbiome. 3:10. doi: 10.1186/s40168-015-0070-0, PMID: 25825673 PMC4378022

[ref67] Yang C. C. Wu C. H. Lin T. C. Cheng Y. N. Chang C. S. Lee K. T.. (2021). Inhibitory effect of PPARgamma on NLRP3 inflammasome activation. Theranostics. 11, 2424–2441. doi: 10.7150/thno.46873, PMID: 33500734 PMC7797672

[ref68] Youngster I. Mahabamunuge J. Systrom H. K. Sauk J. Khalili H. Levin J.. (2016). Oral, frozen fecal microbiota transplant (FMT) capsules for recurrent *Clostridium difficile* infection. BMC Med. 14:134. doi: 10.1186/s12916-016-0680-9, PMID: 27609178 PMC5016994

[ref69] Zemljic M. Rupnik M. Scarpa M. Anderluh G. Palu G. Castagliuolo I. (2010). Repetitive domain of *Clostridium difficile* toxin B exhibits cytotoxic effects on human intestinal epithelial cells and decreases epithelial barrier function. Anaerobe 16, 527–532. doi: 10.1016/j.anaerobe.2010.06.010, PMID: 20620216

[ref70] Zhao J. Zhao R. Cheng L. Yang J. Zhu L. (2018). Peroxisome proliferator-activated receptor gamma activation promotes intestinal barrier function by improving mucus and tight junctions in a mouse colitis model. Dig. Liver Dis. 50, 1195–1204. doi: 10.1016/j.dld.2018.04.016, PMID: 29891333

